# An empirical study of physical activity and sports affecting mental health of university students

**DOI:** 10.3389/fpsyg.2022.917503

**Published:** 2022-09-02

**Authors:** Lu Congsheng, Sumaira Kayani, Amna Khalid

**Affiliations:** ^1^School of Education, Shaanxi Normal University, Xi'an, Shaanxi, China; ^2^Haian Senior School of Jiangsu Province, Nantong, China; ^3^Department of Education, Zhejiang Normal University, Jinhua, Zhejiang, China; ^4^College of Medicine, University of Sharjah, Sharjah, United Arab Emirates

**Keywords:** physical activity, sports activity, mental health, Malaysia, SEM-PLS

## Abstract

Physical activity (PA) and sports are extremely essential elements for physical and mental health among adolescents. Around 30% of 16 years old and above in Malaysia have mental health issues. For this purpose, 512 university students from Malaysia were surveyed through social media, with 74% response rate. Structural equation model partial least square (SEM-PLS) was used to examine the effect of PA and sports on mental health. The results revealed that both PA (*b* = 0.402, *p* < 0.001) and sports (*b* = 0.330, *p* < 0.001) significantly predict mental health among university students. The model explained 35.8% variance in mental health. The study suggests that PA and sports activity need to be promoted among university students to control prevailing mental health issues in adolescents.

## Introduction

Mental health is regarded as the psychological and emotional well-being of a person. It is reflected in the positive functioning in life based on the interaction between social and biological factors (Chow and Choi, 2019; Easterlin et al., 2019). The World Health Organization has defined mental health as “a state of well-being in which the individual realizes his or her abilities, can cope with the normal stresses of life, can work productively and fruitfully, and can make a contribution to his or her community” (Chow and Choi, 2019). However, the study conducted by Pascoe et al. (2020) has highlighted that mental disorders are expected to become a leading reason for causing disability among young people in developed countries. Hence, the promotion of mental health and the prevention of mental disorders is paramount.

Several types of research have indicated that physical activity (PA) and sports play a major role in improving an individual’s mental health at all stages of life (Biddle et al., 2019; Tiaotrakul et al., 2019; Tamminen et al., 2020; Teychenne et al., 2020). PA can be spontaneous (leisure, work, or transport) or organized (physical exercise). Main purpose of physical exercise is to improve the health and physical capacity of an individual. The bio-psychosocial (BPS) model confirms that PA and exercise are among the best coping strategies for mental health problems (Di Benedetto et al., 2010; Di Benedetto, 2015; Poirel, 2017). Physical training is concentrated towards increasing maximum performance and capacity. On the other hand, physical inactivity is a sedentary behavior in which body movement is absent. Physical inactivity leads to increased risk of poor health, which can affect the well-being of an individual (Malm et al., 2019).

Sports is another important activity that contributes to the mental health of youth. The importance of sports among the students is valuable and goes beyond PA. The stress level of college students is higher than the other age groups, which can adversely affect their mental health. Stress is usually caused by academic pressures, which can lead to terrible effects and further results in future failure. However, sports has played a drastic role in reducing the students’ stress levels and increasing happiness and psychological well-being (Judge, 2018).

Malaysian students have been particularly observed to have poor mental health. The COVID-19 pandemic had a further adverse effect on mental health of individuals leading to stress and depression. It has limited the gatherings for PA and sports. During the pandemic, 30% of Malaysian people aged 16 years or older were having mental health issues (Kotera et al., 2021). Furthermore, the youth increased their internet usage due to the protective measures of COVID-19, such as lockdown and social distancing. As a result of high internet usage, health anxiety of the youth is increased (Khodabakhsh et al., 2021). Due to the increasing concern of mental health issues among Malaysia’s youth and the consequences that would endanger the future of the youth and country, it is critical to formulate effective strategies for controlling the prevalence of mental health issues. In this regard, the following research is conducted to evaluate the effectiveness of PA and sports on mental health. This also provides an opportunity to fill the literature gap by studying how PA and sports can positively influence youth’s mental health in Malaysia, as there is no recent study conducted in this context. The significance of this research lies in aiding the government and educational institutes in developing intervention programs based on PA and sports for improving the mental health of the youth in Malaysia.

## Literature review

Mental health is an important area of research where scholars and researchers prioritize enhancing health and achieving equity in health for the entire people throughout the globe (Castillo et al., 2019). Mental health is defined as a state in which the individual can realize his/her capabilities while also coping with the normal stress of life and can work productively. Mental health comprises multiple cognitive and affective components, including happiness, enjoyment and pleasure, and having a purpose, meaning and fulfilment (Silove et al., 2017; Bratman et al., 2019). However, a major concern for mental health is mental illness, which reflects the occurrence of cognitive, affective and behavior problems. Therefore, there is an increased attention to research towards preventing mental illness that can adversely affect mental health (Arango et al., 2018; Bratman et al., 2019).

Furthermore, the importance of preventing the mental health problems has become critical across the globe during the COVID-19 pandemic. World health organization has reported a great concern regarding mental health and psychological consequences of the pandemic (Kumar and Nayar, 2021). An increased rate in mental illness has been reported in developing countries. Mental health professionals and psychologists have speculated that the pandemic is likely to further impact mental health of the people where the cases of depression, self-harm, suicide would increase. Kotera et al. (2021) have highlighted that the youth, particularly university students, face poor mental health, resulting in a negative attitude. The researchers estimated that around 30% of the people aged 16 years or orders are faced with mental health issues. In addition to this, the research conducted by Khodabakhsh et al. (2021) also demonstrated that the COVID-19 pandemic has a major influence on the mental health of individuals resulting in stress and depression. Furthermore, due to lockdown and other protective protocols, the youth in Malaysia is increasingly using internet where it is expected to increase health anxiety and lover PA.

Physical activity is considered an important predictor of health, especially among the youth (Júdice et al., 2017) and is used as a preventive measure against mental illness. PA is defined as the bodily movement by the skeletal muscles that requires the expenditure of energy. PA is reported to have an antidepressant influence on individuals. Exercise is a subset of the PA which is a planned and structured activity for enhancing physical fitness that involves running or weight training. Furthermore, PA also has psychological benefits that may positively influence mental health (Kandola et al., 2019; Ozemek et al., 2019). Based on the BPS model (Di Benedetto et al., 2010; Di Benedetto, 2015), numerous empirical studies (McDowell et al., 2017), and systematic reviews (Mikkelsen et al., 2017; Stubbs et al., 2017) confirm PA as a predominant predictor of mental health. Therefore, PA is reflected as a key strategy for improving the individual’s mental health and well-being benefits. PA comprises structured, supervised exercise programs, active commuting, domestic activities, and leisure physical activities (Rebar and Taylor, 2017). Regular PA is a key health behavior from the public health perspective due to its remarkable effect on health. PA has the potential to prevent mental health disorders and issues that comprise depression and anxiety. Several studies were conducted on the benefits of PA on mental health during COVID-19 (Callow et al., 2020; Lesser and Nienhuis, 2020; Shahidi et al., 2020). The studies highlighted the importance of PA during the COVID-19 quarantine, where the activity appears to have an antidepressant and anxiolytic effect. However, these studies did not recruit young people in Malaysia. Therefore, based on the literature analysis and the gap, the following hypothesis is developed based on PA.

H1: Physical activity has a significant and positive influence on the mental health of youth.

Sports is reflected as another preventive strategy for mental health problems. It is defined by athletic games institutionalized in several ways. Sports is a game that involves physical exertion and skills in which an individual or a team competes for entertainment (Jukic et al., 2020). However, the study conducted by Malm et al. (2019) has demonstrated that although sports enhances the physical as well as the psychological health of individuals, it may adversely affect mental health due to the risk of injury, burnout, and eating disorders. The negative impact is commonly observed among the elite players as they are required to perform at maximum to get the desired outcomes. As a result, this can cause a negative effect on the mental health of the elite-level players. On the other hand, Eigenschenk et al. (2019) indicated that outdoor sports can create positive benefits for mental health as it can cure the mental health problems. Furthermore, engagement in sports positively affects general health and psychological ability by increasing well-being, improving quality of life, and enhancing happiness and life satisfaction. Therefore, based on the literature analysis and the gap, the following hypothesis is developed:

H2: Sports has a significant and positive influence on the mental health of youth.

**Figure 1:**
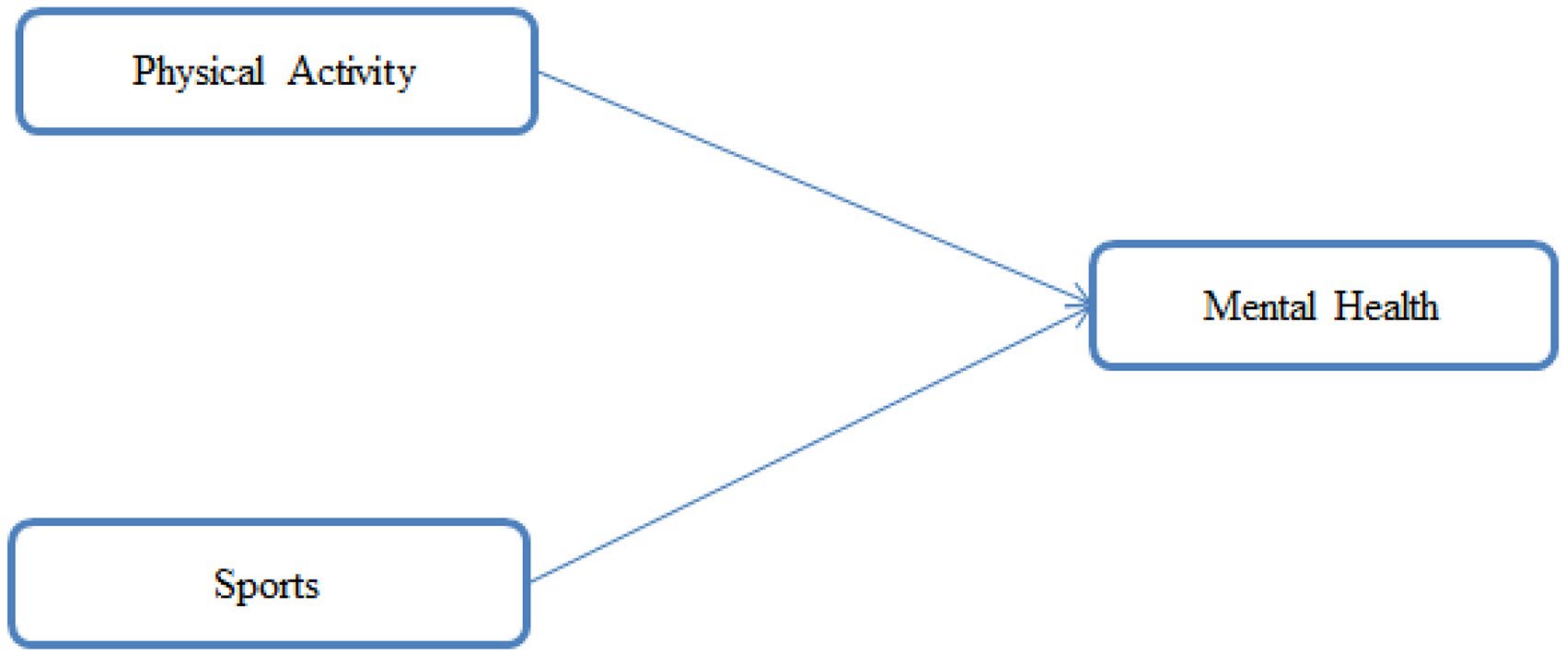
Conceptual framework.

Siefken et al. (2019) evaluated the association between leisure-time PA, anxiety and depression. The study was conducted through a questionnaire survey distributed among the athletes across the globe in which total number of completed surveys was 682. The findings from the research indicated that individuals who met the recommended levels of sports and PA reported lower scores on depression. Furthermore, the lowest depression and anxiety scores were identified among the indoor team athletes. Bell et al. (2019) studied the interrelation between PA, mental well-being and symptoms of mental disorders among young British adolescents. The multivariable regression analysis indicated no strong evidence as to whether PA is connected with better health and well-being. Reigal et al. (2021) examine the interconnection between PA, mood states, and self-rated health in Spain. The results indicated that PA positively effects mental health by improving perception and mood during pandemic lockdown. The review of the literature shows that there is a clear gap in research on relationship between PA, sports and mental health in Malaysian youth. The previous findings reported during lockdown have shown mixed results. Therefore, it is imperative to explore these constructs among Malaysian youth during COVID-19 pandemic. Figure 1 represents the conceptual framework for the present study, aimed at investigating the influence of PA and sports on mental health of the youth in Malaysia.

## Methodology

### Participants

The targeted audience for the questionnaire survey are mainly the adolescents in Malaysia; therefore, the questionnaire is provided to the students that are currently studying in Malaysian universities. The approach for gaining access to the target audience was distributing the questionnaire through the social media platforms such as Facebook, Instagram, and others. The use of the internet has enabled in easily obtaining the data from Malaysian adolescents. As for the sample size, the total number of questionnaires was distributed among 700 adolescents, where 512 participants have provided the complete data. Response rate was 74%. That may be because the study was conducted during COVID-19 pandemic situation in the country, and the universities were not opened. The student may have adopted casual attitude due to study from home. Hence, most of the students did not bother responding the survey. Characteristics of participants are given in table below (see Table 1).

**Table 1:** Characteristics of participants.

Characteristics = (512)	Frequency (*n*)	Percentage (%)
Gender		
Male	239	46.68
Female	273	53.33
Age		
18–20 years old	156	30.47
21–23 years	149	29.10
24–26 years	207	40.43
Rurality/urbanicity		
Rural	365	71.29
Urban	147	28.71
Socioeconomic status		
Lower class	188	36.72
Middle class	287	56.05
Upper class	37	7.22

### Instruments

To investigate the role of PA and sports on mental health, the researchers have designed a survey used in the previous research (Snedden et al., 2019; Appelqvist-Schmidlechner et al., 2020).

The measure of mental health contained four items: I’ve been feeling optimistic about the future,” “I’ve been feeling relaxed,” “I’ve been dealing with problems well,” and “I’ve been able to make up my own mind about things.” Initially, there were seven items out of which 3 were eliminated during validation process. The items were rated on a 5-point Likert scale from 1 (none of the time) to 5 (all of the time) (Appelqvist-Schmidlechner et al., 2020).

For PA, self-reported leisure time PA was determined from responses to a single question. Think of the last 3 months and consider all leisure-time PA that lasted at least 20 min per session, and answer: “Which of the following definitions best describes your leisure time PA habits?” Initially, there were 6 response categories which were changed to 4 in the current study: 1 = less than once a week, 2 = no vigorous activities, but light or moderate PA at least once a week, 3 = brisk PA once a week, and 4 = vigorous activity at least four times a week. For further analysis, responses were binned into four categories indicating inactive (response 1), low (response 2), medium (responses 3), and high (response 4) leisure time PA (Fogelholm et al., 2006; Appelqvist-Schmidlechner et al., 2020).

Further, Self-assessed sport activity level was measured through four items adapted from previous study (Snedden et al., 2019). The students were asked to report their “athletic activities and daily work out” rated on 5-point Likert scale from 1 (do not perform at all) to 5 (perform daily). All tools were validated before use in Malaysian setting. The results of validation process are given in the results section.

### Analysis techniques

The analysis of the gathered data is mainly conducted through statistical analysis with the support of the statistical software, SmartPLS. The process for using the SmartPLS was first to convert the questionnaire data to numerical values and was further coded to SmartPLS for generating results. There are mainly two techniques that are applied from software for revealing the results and findings. The first technique applied with the software is the confirmatory factor analysis, also referred to as CFA. The technique aims to evaluate the validity and reliability of the dataset in which the reliability and convergent validity are assessed along with the evaluation of the discriminant validity. The second technique applied is structural equation modelling, in which the cause and effect of the identified variables are assessed.

## Results

### Descriptive statistics and correlation among study variables

Table 2 presents descriptive statistics and correlation among study variables. It is shown that there is strong positive association among all study variables. However, the mean level of PA is higher than that of sport activity.

**Table 2:** Descriptive statistics and correlation among study variables.

Variables	Mean	SD	Mental health	Physical activity	Sport activity
Mental health	3.870	2.26	1	0.68	0.80
Physical activity	4.023	2.37		1	0.74
Sport activity	3.692	2.08			1

### Reliability and validity

In the structural equation model and partial least square (SEM-PLS), it is deemed a prerequisite to determine the reliability and validity of the constructs. In an empirical study, multiple constructs are used to measure the concepts and theories to be tested, and each of the constructs has different components or indicators representing the construct. For instance, the first construct of the study was the measurement of mental health, which has four indicators, and similarly, PA and sports activity are two different constructs with four indicators each. In such a setting, scholars (Amaro et al., 2015; Hair et al., 2017) have been using the outer loadings, also known as factor loadings, which indicates the variance being predicted each indicator. As per Hair et al. (2017), the factor loading value needs to be greater than 0.7 to confirm the significant contribution of the factor that it sufficiently contributes to the construct to measure the concept. However, if the factor loading value remains below the given threshold, the indicator has been dropped from within the construct since its presence does not contribute significantly. Similarly, Table 3, which depicts the indicators along with the factor loadings, shows that all indicators within the study have factor loading greater than 0.7, suggesting that all indicators contribute sufficiently to the constructs, and all indicators can be retained within the model.

**Table 3:** Factor loading, reliability, and validity.

Indicator	Outer loading	Cronbach’s alpha	Composite reliability	Average variance extracted (AVE)
MH1	0.888	0.918	0.942	0.803
MH2	0.921			
MH3	0.880			
MH4	0.895			
PA1	0.820	0.886	0.921	0.744
PA2	0.872			
PA3	0.899			
PA4	0.856			
SA1	0.793	0.821	0.883	0.654
SA2	0.843			
SA3	0.884			
SA4	0.704			

Furthermore, the reliability and validity of the constructs have also been determined within the study. In accordance with the definition of Janadari et al. (2016), reliability refers to the internal consistency within the responses gathered through the construct, and validity refers to the extent to which a construct measures for what it has been designed to measure. To further explore, the opinion of Akter et al. (2017) can be presented where authors have stated that reliability reveals the effectiveness of the construct that whether the constructs used in the survey are producing consistent results or not? It means does it work in an intended way or not? In addition, the concept of validity is also referred to as convergent validity, which represents the extent to which constructs that must be related are related. Meanwhile, Hair et al. (2017) shed light on the convergent validity as that it tells do indicators within the constructed measure the same thing which it must measure actually.

For reliability of the constructs, two empirical techniques have been used; Cronbach’s alpha and composite reliability. Both techniques are being used to assess the internal consistency within the responses to see if constructs produce similar results. Meanwhile (Hair et al., 2017), state that for constructs to be labelled as reliable, the alpha value needs to be above 0.7 in Cronbach’s alpha and Composite reliability results. However, to determine the compliance of constructs with the convergent validity, average variance extracted (AVE) has been used as a technique. Amaro et al. (2015) deem its prerequisite for assuming convergent validity that the AVE needs to be greater than 0.5. Table 2 reveals that Cronbach’s alpha and value of composite reliability are greater than the suggested threshold of 0.7 indicating that there is internal consistency within the responses, which supports the statement that constructs have produced consistent results and have worked in a way they were designed. Similarly, referring to convergent validity, the AVE for all constructs remains greater than 0.5, which supports the statement that constructs which must have been related are related.

The analysis of CFA reveals that the data used in the following study is reliable and valid to be used for further study, and it would also produce valid and consolidated unbiased results.

### Discriminant validity

Discriminant validity is a technique to determine how many constructs are accurate in conceptual measurement. Hence, to confirm the accuracy of these measures, the concept of discriminant validity has been applied, which determines the extent to which the constructs that should not be related must be unrelated. To further explore the concept of discriminant validity, Hair et al. (2017) sheds light that a construct measures a unique concept or theory and, in this way, each of the measurements of the construct should be distinct and must not be related. In this regard, Heterotrait-Monotrait (HTMT) ratio has been used to determine the conceptual accuracy of the constructs and result are illustrated in Table 4.

**Table 4:** Discriminant validity.

Heterotrait-Monotrait ratio (HTMT)	Mental health	Physical activity
Physical activity	0.560	
Sports activity	0.531	0.381

In accordance with the study conducted by Hair et al. (2017), it is deemed a prerequisite that the value of the HTMT ratio needs to be below the threshold of 0.9 to confirm compliance with the discriminant validity. Meanwhile, the value of ratio as illustrated in Table 1 also meets the criteria outlined by the authors, which confirms that there is also discriminant validity.

### Model specification

In empirical models, the focus of scholars’ remains with the coefficient of determination which is also referred to as *R*^2^, indicating the predictive strength of the model. The model of the study is depicted in Figure 1.

In the current model, the *R*^2^ remains to be 0.358 suggesting that 35.8% variance of mental health has been estimated by PA and sports activity, citing that 74.2% variance remains residual of the empirical model (see Table 5). This implies that residual can be estimated, or the predictive power of the existing model can be strengthened by the inclusion of other factors and variables into the model. In addition, the adjusted *R*^2^ is 0.356, which is less than *R*^2^ and supports the statement that inclusion of the factors and variables would further improve the model’s predictive power.

**Table 5:** Model specification.

*R* ^2^	*R* ^2^	Adjusted *R*^2^
Mental health	0.358	0.356

### Path coefficients

Table 6 provides the path coefficients, where it is evident that one unit of the change into the PA and sports activity will also bring a change of 0.402 [*P* value 0.000] and 0.330 [*P* value 0.000], respectively. This suggests a positive and statistically significant impact of PA on the mental health of youth in Malaysia, and this implies that prevalence of PA among the youth can be one of the important factors to keep them away from mental health issues. Similarly, the effect of sports activity on mental health has also been found positive and statistically significant, suggesting that the involvement of the youth in sports can help them overcome mental health issues. As a result, the study’s hypotheses are accepted that PA and sports activity have a significant and positive impact on the youth’s mental health in Malaysia. Therefore, it can be asserted that PA and sports activity needs to be promoted among the university students of Malaysia in order to control prevailing mental health issues within the youth and secure a better future for the generations to come.

**Table 6:** Path coefficient.

	Original sample	*t* statistics	*P*
Physical activity → mental health	0.402	9.102	0.000
Sports activity → mental health	0.330	6.836	0.000

## Discussion

Contributing to the literature, the current study validates a conceptual model reflecting the association of PA and sports with students’ mental health. The hypothetical model in our study suggests the importance of a theoretical framework predicting mental health in students. Further, our research shows novelty for authenticating BPS model in Malaysian setting.

The findings presented that PA was positively associated with mental health of students. Past research exhibited the similar results regarding the link of PA with mental health of university students in different cultures (Kayani et al., 2020a,b, 2021). BPS model of Di Benedetto also confirms the positive association of exercise and mental health (Di Benedetto et al., 2010; Di Benedetto, 2015). Moreover, PA has been shown to have an antidepressant effect, implying that it can be used as a mental health prevention strategy (Kandola et al., 2019). Moreover, Kandola et al. (2019) also argues that PA offers mental health benefits that may positively impact mental health. Furthermore, Clough et al. (2016) claimed that engaging in 15 min of PA every day reduces the risk of sadness and anxiety by 26%. Adopting a regular fitness routine lowers the chances of relapsing. PA generates various favorable changes in the brain, making it a reliable therapeutic for psychiatric illnesses. The generation of endorphins contributes to a sense of relaxation and well-being. These activities also diverge negative emotions and other factors that contribute to depression and stress. That is, PA significantly enhances mental well-being among students.

Furthermore, the study has found sport performance positively predicted mental health. In association with previous research (Kayani et al., 2020a,b, 2021), increased sport and exercise enhances psychological well-being. In addition, sports are represented in another mental health prevention plan, where they are described as athletic games that are organized in various ways. Jukic et al. (2020) stated that sports involve physical effort and talent in which individuals or teams compete against one another for enjoyment. Eigenschenk et al. (2019) has indicated that outdoor activities provide good advantages for mental health through curing mental health issues. By promoting well-being, improving life, and boosting pleasure and life happiness, participation in sports has a favorable influence on overall health and mental ability. In addition, Siefken et al. (2019) conducted a study to see if sports impact mental health and analyze the link between leisure-time physical exercise and depression and anxiety. In sum, the study provides evidence for the importance of PA and sport participation in Malaysian society. Apart from this, the study portrays unidirectional relationships among all study variables. Two way associations are hence recommended to get more insight into the study variables.

## Research implications

The study’s findings imply that regular PA and sport performance would contribute positively towards mental well-being. These findings are in agreement with the BPS model (Di Benedetto, 2015) in relating exercise PA with mental health. The current study provides significant piece of knowledge portraying PA and sport as protective factor for mental health issues in the context of Malaysian university students.

The study also contains practical implications. The study has called our attention to the importance of PA and sport in promoting mental well-being among university students. The current research has identified that PA and sport play a valuable role in enhancing mental health of students. Therefore, the results suggest encouraging physical activities and sport performance among university students in mental health promotion and intervention initiatives. The research also recommends that institutions may consider teachers’ professional development in the domain of encouraging PA and sport participation. Future research in other cultures with the combination of other contributing factors affecting mental health may assist to better understanding of the phenomenon. Further intervention studies aiming at the promotion of well-being through a PA and sport could be taken into consideration.

## Limitations and future research directions

This research describes several significant practical and theoretical implications, but some limitations need to be addressed in upcoming studies. For instance, this research has tested the model based on empirical evidence, which might be criticized for common method bias. Therefore, we suggest a qualitative approach and an in-depth interview to be conducted in future studies. Another limitation is that the research has been conducted in Malaysia by collecting data online from only one region of the country. This might cause sampling bias, and the results might not be generalized to other parts of the country. To get more generalizable results, samples from other cities and provinces of Malaysia may also be taken for future research. Further, the research is based on self-report measures, which may lead to inflated relationships. Future research could be conducted with experimental designs. Moreover, the sample in the present research is a relatively homogenous group of university students. Taking the heterogeneous sample may generate different findings.

In addition, the teachers, educationists and health practitioners should take the importance of PA and sport to promote mental well-being among university students. A recent study has provided a qualitative approach to develop PA leaders using a contemplative education approach to promote wellness among the elderly (Tiaotrakul et al., 2019). The same approach could also be adopted for training the leaders guiding students to perform PA and sport.

## Conclusion

This study reports on research on the effect of PA and sport activity on mental health of Malaysian university student. Underpinning this research was the BPS model of exercise, mental and physical health. This study’s research model was based on the evidence gathered from 512 students enrolled in different disciples of in Malaysian universities. After analyzing data in the structural model using PLS, we found that both PA and sport activity significantly predicted mental health of university students. However, the magnitude of the effect size of PA is more than sport performance. Therefore, our findings revealed that PA and sport performance contribute to sound mental health in Malaysian context. The research suggested that students who participate in PA and sport would have better mental health.

## Data availability statement

The raw data supporting the conclusions of this article will be made available by the authors, without undue reservation.

## Ethics statement

The studies involving human participants were reviewed and approved by Shaanxi Normal University. The patients/participants provided their written informed consent to participate in this study.

## Author contributions

LC wrote the original draft. SK helped in method and data analysis. AK did critical analysis and edited the manuscript. All authors contributed to the article and approved the submitted version.

## Conflict of interest

The authors declare that the research was conducted in the absence of any commercial or financial relationships that could be construed as a potential conflict of interest.

## Publisher’s note

All claims expressed in this article are solely those of the authors and do not necessarily represent those of their affiliated organizations, or those of the publisher, the editors and the reviewers. Any product that may be evaluated in this article, or claim that may be made by its manufacturer, is not guaranteed or endorsed by the publisher.
